# Having less and wanting more: an investigation of socioeconomic status and reinforcement pathology

**DOI:** 10.1186/s12889-021-10430-7

**Published:** 2021-02-25

**Authors:** Amanda K. Crandall, Amanda M. Ziegler, Tegan Mansouri, Jalen Matteson, Emily Isenhart, Autum Carter, Katherine N. Balantekin, Jennifer L. Temple

**Affiliations:** 1 Department of Community Health and Health Behavior, School of Public Health and Health Professions, University at Buffalo, 3435 Main Street, 1 Farber Hall, Buffalo, NY 14214 USA; 2 Department of Exercise and Nutrition Sciences, School of Public Health and Health Professions, University at Buffalo, Buffalo, USA

**Keywords:** Adolescents, Obesity, Reinforcement, Delay discounting, Socioeconomic status, Food insecurity

## Abstract

**Background:**

In the United states obesity and socioeconomic status (SES), or one’s standing in society based on income, education, and/or occupation, are strongly associated. The mechanisms for this relationship may include having high levels of motivation to get food (reinforcing value of food; RRV) and low levels of inhibitory control (delay discounting; DD) which, when combined, is referred to as reinforcement pathology (RP). We sought to examine the relationships among multiple measures of household SES, RP, and age-adjusted body mass index (zBMI) among adolescents.

**Methods:**

These data were collected as part of ongoing longitudinal study of risk factors for obesity in 244 adolescents. The adolescents and one parent/guardian had height and weight measured and completed surveys. The adolescents completed an adjusting amount DD task and a computer-based RRV task. Analyses consisted of correlations among measures of SES and RRV, DD, and BMI z-scores. In the case of significant associations, multiple regression models were created with theoretically informed covariates.

**Results:**

Household income, parent/guardian education, parent/guardian occupation, and food insecurity status were all related to one another. Among the adolescents, a significant portion of the variance in RRV was accounted for by household income after controlling for covariates. For DD, it was parent/guardian education that was most associated after controlling for covariates.

**Conclusion:**

When low income and low parent/guardian education occur together, there may be an increased risk of RP. Separately, food insecurity was predictive of higher parent/guardian BMI. Future research should continue to explore the effects of low income and parent/guardian education on RP among youth by examining them over time.

**Supplementary Information:**

The online version contains supplementary material available at 10.1186/s12889-021-10430-7.

## Background

Obesity is a complex disease that is influenced by multiple social, environmental, behavioral, and developmental factors [6, 26]. Disparities in income, education, and environments play a significant role in obesity risk [6]. One significant factor contributing to health disparities in obesity, both among adults and developmentally among children, is socioeconomic status (SES) [26]. SES in the United States is a confluence of related factors, including income, educational attainment, wealth, neighborhood resources, and career prestige, that work independently and interactively to advantage certain sectors of society over others [28]. Those with fewer economic resources are at a higher risk for food insecurity, which is a consistent worry or concern about one’s ability to obtain an adequate supply of nutritious foods [19]. Both SES and food insecurity are independently associated with obesity [19] and may directly impact eating behavior, as periods of uncertainty surrounding access to food may promote overeating when food is available and dissociate eating from physiological hunger and fullness cues [9].

Socioeconomic status may exert an influence on eating behavior by way of the motivational and inhibitory centers of the brain [27]. Having high levels of motivation and low inhibitory control has been conceptualized as reinforcement pathology [3]. Motivation to obtain food, or the relative reinforcing value (RRV) of food, is measured by determining the amount or work that an individual will invest to access food [10]. Higher RRV of food predicts both energy intake and obesity [10]. By contrast, inhibitory control works to counteract high levels of motivation [22]. One aspect of this is delay discounting (DD), which is a state of highly valuing smaller immediate rewards over larger, long term rewards. Increased energy intake and obesity have been linked to higher DD (i.e. a greater valuation of immediate rewards) [1] independently, as well as interactively with RRV (i.e. reinforcement pathology; RP) [30].

Emerging evidence suggests that SES may increase reinforcement pathology. Food restriction and deprivation, and even deprivation in the past, has been shown to increase RRV of food [24, 29]. Additionally, laboratory research has shown that humans’ desire for money and food tend to overlap, such that hungry adolescents desire more money and those primed with financial dissatisfaction tend to consume more energy [2]. Past research has shown that RRV is related to both household income and education level in adults [20]. Finally, experimental evidence has suggested that food insecure adults may experience an increase in their RRV of food in response to financial losses [7]. DD is inversely associated with SES in adults [25]. Further, adults who grew up in low-SES households tend to respond to scarce resources with more impulsivity [13]. Children living through more adverse childhood experiences related to poverty have more difficulty delaying gratification [11]. When taken together, this evidence suggests that low SES and food insecurity may increase the RRV of food among adults, and poverty may increase DD in children and adults. Thus, moving down the socioeconomic ladder in the United States may increase one’s risk of reinforcement pathology.

Although these relationships have been shown independently, previous research has not examined them in the same sample, and few have examined the effect of poverty and food insecurity among adolescents. Adolescence is a developmental period in which reward sensitivity is high and self-control pathways, such as DD, are poorly developed [4]. Thus, adolescence may pose a sensitive period for the development of RP. The current study examines baseline data from an ongoing longitudinal study of obesity risk/protective factors in adolescents [33]. We hypothesized that parent/guardian education, household income, and household food insecurity would be interrelated and related to parent/guardian BMI and adolescent zBMI. For the investigation of RP, we first examined RRV and DD separately. We hypothesized that household income and parent/guardian education would be inversely related to DD in the adolescent. We hypothesized that adolescent level food insecurity would be positively related to the adolescents’ RRV of high energy density (HED) food. Finally, we hypothesized that a composite score of our SES variables would account for a significant amount of the variance in overall RP scores in this sample. We also examined the relationship between food insecurity and RRV of low energy density (LED) food, but started with no directional hypotheses due to the lack of previous work in this area. This study was approved by the University at Buffalo Institutional Review Board (MOD00008052).

## Methods

### Study participants

The data presented here are from the baseline phase of a 2-year longitudinal study assessing behavioral predictors of weight change across adolescence [33]. Adolescent participants were 12 to 14 years old, without current underweight or obesity, and reported a willingness to participate in 9 appointments over 2 years. Adolescents with obesity were excluded from the study because the primary purpose of the overall longitudinal study was to examine obesity risk factors. Exclusion criteria included any reported diagnosis of an endocrine disorder, taking medication affecting appetite/weight, and/or an inability to walk on a treadmill and/or lift arms above head (due to a physical activity component of the parent study). To be included in the study, they also needed to be willing to eat at least one high- and one low-energy dense food for 2 weeks that they rated with moderate or higher liking and consumed less than 4 times per week in their usual diet. A parent/guardian accompanied the participant to the initial appointment and responded to several questionnaires (described below) in order to gather more data about the adolescent’s life and health. Researchers requested that whichever parent/guardian was most responsible for food decisions in the household accompany the adolescent to the appointment.

### Study procedures

The baseline phase of this study included 5 laboratory visits over the course of approximately 8 weeks. The first appointment was a screening visit, followed by 4 food exposure visits. The study timeline varied slightly between participants depending on the time between the screening appointment and the food exposure appointments (described below). Adolescents, along with one parent/guardian, visited the laboratory for an initial appointment, during which the height and weight of both parent/guardian and adolescent were measured. Adolescents rated their liking, frequency of consumption, and willingness to eat six high energy density (HED; > 4 kcal/g) and six low energy density (LED; ≤ 1 kcal/g) snack foods. They were also asked to complete a battery of electronic surveys related to food insecurity and child eating behaviors, administered via Survey Monkey. Adolescents completed the hypothetical DD task with a trained research assistant while the parent/guardian completed demographic and food insecurity questionnaires. Adolescents were eligible for the study at the end of this appointment if their BMI z-score was between − 1.5 and + 2.0 (i.e. those without underweight or obesity according to the World Health Organization) [16].

During the baseline HED and LED snack visits, adolescents arrived in the laboratory 2 h fasted. They rated appetite sensations of hunger, thirst, liking, pleasantness, and wanting of their assigned food before consuming a preload granola bar (130 kcal). During a minimum of 15-min waiting period, we conducted a same-day dietary recall to verify no food or drink was consumed (other than water) during the fasting period. Appetite sensations were rated once again before engaging in the RRV task for the snack vs. alternative reinforcer. After the RRV task, adolescents ate and/or used the activity time as they wished. Following the baseline HED and LED appointments, participants were sent home with food to consume every day for 2 weeks. RRV measurements for each food were then reassessed. The order of food appointments (HED vs LED) was counter-balanced and participants had a minimum of 1 week between food exposures (HED vs LED). Only the baseline food appointments will be examined in this study. Please see Temple and colleagues [33] for full study description and baseline results.

#### Relative reinforcing value of foods (RRV)

To measure the RRV of HED and LED foods, adolescents completed a standardized task [10]. Adolescents were presented with two computers, and could play for food portions on one and seated activity time on the other. Both the study food and seated activity were chosen during the first appointment, based on self-ratings of available food and activity. The available HED foods were plain milk chocolate candies, miniature peanut butter cups, chocolate chip cookies, plain potato chips, tortilla chips flavored with cool ranch seasoning, and crunchy cheese flavored snacks. The available LED foods were vanilla low-fat yogurt, blended low-fat strawberry yogurt, mandarin orange fruit in 100% juice, diced peaches in 100% juice, diced pears in 100% juice, and plain applesauce. The available seated activities included time to draw or color pictures, solve puzzles and use activity books, and play electronic (non-computerized) games (e.g., Simon or electronic poker).

The RRV task is similar to a slot machine game, as each computer screen showed a set of three different colored shapes, and adolescents had to click the screen to rotate the shapes and get one row of shapes to match. One point was earned each time all shapes matched, and once five points were earned on a computer, adolescents were given either one portion of their chosen study food or a designated amount of time for their chosen activity. Adolescents could only play on one computer screen at a time, but could switch back and forth between screens as they pleased. Each round required more mouse clicks as the participant moved through the task. The difficulty level was based on a progressive ratio schedule, with schedules of reinforcement of 4, 8, 16, 32, 64, etc. Upon completion of the task, adolescents were given time to eat the study food portions they earned and use their seated activity time, separately, if they wished. To calculate an individual score for RRV, area under the curve was calculated based on responses to each schedule or reinforcement for each reinforcer on all laboratory visits. These methods have been shown in the past to reliably measure RRV of food, which is a strong predictor of energy intake in a laboratory setting [10].

#### Delay discounting task (DD)

DD was assessed on the first visit using the adjusting amount DD task. The DD task required adolescents to make choices between an amount of money available immediately or $50 available later. The immediate value was adjusted until it was subjectively equivalent to the later larger amount (starting at $0.50 and progressively increasing to $50). The point at which the immediate value is chosen over the delayed value is known as the indifference point. Indifference points were obtained at 6 delays (1 day, 2 days, 1 week, 2 weeks, 1 month, and 6 months). The rate of discounting, k, was calculated using Mazur’s hyperbolic discounting equation [21, 30]. This method has been validated against real-world monetary choices over delayed time periods [18].

#### Reinforcement pathology (RP)

RP is the interaction of HED RRV and DD. This variable was calculated by multiplying the RRV area under the curve score by DD k, described above. Higher scores reflect greater RP, and represents a particularly powerful risk factor for obesity [3, 30].

### Measures and questionnaires

#### Demographics

A standard demographic questionnaire, which was filled out by the parent, assessed child age and sex, parent/guardian marital status, employment status, educational attainment, total household income, and occupation (See supplemental materials). Parent/guardians who attended the appointment were asked to complete the form for themselves and for other caregivers of the participant. The parent/guardian had the discretion of choosing who, if anyone, they would specify as the secondary caregiver. Most of the parents reported that they were married (76%) and most reported education and occupation for the non-participating biological parent (77%). Demographic information was not collected for additional caregivers.

#### Parent/guardians’ education

The reported educational levels of two caregivers were averaged together to create a composite score of parent/guardians’ education. One missing value was allowed in the calculation, so that single-parent/guardian households were included.

#### Total household income

Parents/guardians reported total household income employment income, government assistance, child support/alimony, and disability. This question offered ranges of income levels from “Under $9999” to “Over $200,00” (Table 1). In order to include this variable in correlation and regression analyses, the midpoint of each income range was used as the value for household income.

**Table 1:** Participant Characteristics

Demographic Variable	Value	N (%)
Adolescent sex	Male	120 (49.4)
	Female	123 (50.6)
Parent education level	High school (Some or completed)	13 (5.3)
	Some college and/or vocational training	35 (14.4)
	Completed college or University (2 or 4-year degree)	100 (41.2)
	Completed Graduate Degree	94 (38.7)
Adolescent Race/Ethnicity	Black/African American	29 (11.9)
	White	186 (76.5)
	Other or more than one race	27 (11.1)
	Hispanic or Latinx	17 (7.0)
Total Household Income	Under $9999 – 29,999	21 (8.8)
	$30,000 – 69,999	58 (23.9)
	$70,000 – 109,999	69 (28.4)
	$110,000 – 179,999	73 (30.1)
	$180,000 – Over $200,000	21 (8.7)
Adolescent BMI	Under/Normal weight	170 (70.0)
	Overweight/Obese	73 (30.0)
Household Food Security Status	Fully Food Secure	188 (77.4)
	Marginal Food Security	22 (9.1)
	Low Food Security	17 (7.0)
	Very Low Food Security	6 (2.5)
Dependent Variable		M (SEM)
Adolescent zBMI		0.41 (0.06)
Parent BMI		29.54 (0.43)
Adolescent HED RRV		3.83 (0.12)
Adolescent LED RRV		3.21 (0.12)
Adolescent DD		−5.74 (0.33)
Adolescent RP		−2.84 (0.45)

*Note.* Data includes all adolescents from first screening appointment. Reported means for RRV, DD, and RP are logged values

#### Parent/guardians’ occupation

Two independent coders scored the parent/guardian-reported occupations on a prestige scale of 1 to 9 (1 = Farm laborers/Menial Service Workers, 9 = Higher Executive, Proprietors of Large Businesses, and Major Professionals) using census codes [5]. Mismatches between the two coders were resolved by the study coordinator and PI. Students, homemakers, and those who were unemployed were treated as missing. As with parent/guardian education, the scored values for parent/guardian occupation were averaged together, with missing values allowed, creating a composite score of parent/guardians’ occupation [5].

#### Food insecurity

Parent/guardians answered questions about their own feelings of food insecurity as well as the overall household food insecurity levels using a 17-item subset of the USDA Household Food Security scale [17]. Questions included, for example, “I/We worried whether our food would run out before I/we got money to buy more,” and “I/We relied on only a few kinds of low-cost food to feed the child because there wasn’t enough money for food.” Responses could be, “Often true in the last 12 months, sometimes true in the last 12 months, never true in the last 12 months, I don’t know, or prefer not to answer.” Affirmative responses were summed to create total scores for the adult items (possible score of 0–10), which were then broken into the standard categories of food insecurity (0 = Food secure, 1–2 = Marginal food security, 3–5 = Low food security, 6–10 = Very low food security). Likewise, affirmative answers on the full household questionnaire (possible score of 0–17) were summed and then broken into the standard categories of household food security (0 = Food secure, 1–2 = Marginal food security, 3–7 = Low food security, > = 8 = Very low food security). The adult scale was used for analyses that only included parent/guardian data. All analyses that included adolescent data included the full household scale. This measure also assessed current and past receipt of benefits from the Supplemental Nutrition Access Program (SNAP); however, this question is not used in the scoring of food insecurity [17]. The Household Food Security Scale has been validated for both the measurement of population and individual level food insecurity levels [12] (α = 0.88).

#### Dietary restraint

Dietary restraint was assessed using a version of the Dutch Eating Behavior Questionnaire (DEBQ) modified for use in children and adolescents. The modified DEBQ contains fewer questions than the original and was modified to be more appropriate for children and adolescents. Response options include: never, sometimes, and very often. Dietary restraint was operationalized as the total score of the items on this measure. This measure has been shown to be a valid measure of dieting awareness in children and adolescents [15] (α = 0.80).

#### Body mass index (BMI)

Weight (kg) and height (m) were used to determine the adults’ BMI using the standard equation: kg/m^2^. For children and teens, BMI is age and sex specific, yielding a z-score (zBMI) based on a standard population, which reflects growth patterns in the United States. In this way, zBMI is a relative number for children and teens at a specific age [16].

#### Appetite sensations

During each of the four food appointments, adolescents rated their feelings of hunger, thirst, food liking, and food wanting for the food that they would be working for on the RRV task. Ratings were completed using a 100 mm visual analogue scale. This scale has been used in prior studies to examine current appetite sensations [7, 32].

### Analytic plan

Sample size was determined for the research questions of the parent study [33]. In order to determine if this sample was adequate for the current research question, we examined the relationship between food insecurity status and the relative reinforcing value of food among adults in the control condition of a previous experimental study in our laboratory [7]. After controlling for the same covariates in the current study, this relationship had an R^2^ of 0.24. With an alpha of 0.05, and a power of 0.80, statistical significance could be achieved with a total of 43 participants.

Each dependent variable was checked for skew by visual examination of the histogram. RRV, DD, and thereby RP had a positive skew and were log transformed after converting 0 values of RRV and DD into 1 and .0000001, respectively. For correlations and regression analyses, linearity of each relationship was checked by visual examination of a scatterplot. Visual examination was also used to assess the normality of the residuals in each regression analysis. Multicollinearity was examined using variance inflation factors (all < 2). Finally, heteroskedasticity was assessed for each regression model using the Bruech-Pagan test. In the case of a violation of heteroskedasticity, robust standard errors were calculated using the PROCESS macro for SPSS [14].

Our first hypothesis of the interrelated nature of the SES variables was tested by assessing Pearson product moment correlation coefficients. For the remaining hypotheses, correlation coefficients were examined first, and in the event of significance, multiple regression models were created examining the same relationship while controlling for covariates. In the case of BMI/zBMI, minority status was investigated as a potential covariate for both parent/guardians and adolescents using One-way ANOVA. Age and sex were also used as covariates in the BMI/zBMI models. Covariates for the DD analysis included adolescent age and sex. For the examination of RRV (both LED and HED), covariates were based on previous research and included self-rated hunger, liking of the study food, reinforcing value of the alternative reinforcer (to control for high responders), and dietary restraint. For our final, unified model, predicting RP, covariates included significant predictors from the HED RRV and DD models.

## Results

### Sample characteristics

The sample consisted of 243 (51% females) adolescents aged 12–14 years. Sample characteristics are reported in Table 1. In most cases, the biological mother (*N* = 187) was the parent/guardian who accompanied the adolescent to the screening appointment. Of the original 243 adolescents who attended the first appointment, 239 met the zBMI eligibility criteria at the end of the screening appointment. Of these, 226 appropriately completed the HED baseline appointment. Six did not return for the HED baseline visit and seven deviated from the experimental protocol. For the LED baseline, 223 completed this appointment. Nine did not return for the appointment and seven deviated from the experimental protocol. Deviations from the experimental protocol included eating within 2 h of the scheduled appointment or being served the wrong food during the appointment that was not well liked or novel enough to meet inclusion criteria. A one-way ANOVA showed that the families without complete RRV data for either LED or HED foods did not differ in terms of zBMI, household income, or parent education compared with those who completed these appointments. Chi-Squared tests showed no differences between adolescents with complete versus incomplete RRV visit data in terms of sex or household food insecurity status.

Within the final (*N* = 243) sample, 27 (10%) households were determined to be food insecure (assessed in parent/guardian). A majority of the adolescent sample (*n* = 186) was white (reported by parent) with the next most represented race being Black/African American (*n* = 29). Forty-five families (19%) had a household income of $50,000 or lower.

### Confluence of SES variables

As predicted, household SES, household income, household food insecurity, parent/guardian level food insecurity, current SNAP participation, and minority status of both parent/guardian and adolescent were all related to one another (all *p* < .05). Food insecurity, SNAP participation, and adolescent minority status were all positively related to one another and negatively related to parent/guardian education, parent/guardian occupational prestige, and total household income (See Table 2).

**Table 2:** Interrelationships between Socioeconomic Status, Food Insecurity, and Minority Status

	2	3	4	5	6	7	8
	Min-A	Edu-P	Occ-P	Inc-P	HH-P	FI-P	SNAP-P
1. Parent Minority Status (Min-P)	.80***	−.17**	−.20**	−.28***	.37***	.36***	0.17*
2. Adolescent Minority Status (Min-A)	–	−.17**	−.23***	−.30***	.27***	.25***	.18**
3. Parent Combined Education (Edu-P)		–	.55***	.47***	−.33***	−.31***	−.21**
4. Parent Combined Occupation (Occ-P)			–	.46***	−.25***	−.26***	−.20**
5. Total Household Income (Inc-P)				–	−.31***	−.29***	−.37***
6. Household Food Insecurity Score (HH-P)					–	.88***	.38***
7. Parent Food Insecurity Score (FI-P)						–	.31***
8. Current SNAP receipt (SNAP-P)							–

* *p* < .05 ** *p* < .01 *** *p* < .001

### SES and BMI/zBMI

There was a trend for an effect of race on parent/guardian BMI (F (4, 236) = 2.38, *p* = .052), and the Biracial parents were the highest. There was no effect of race on adolescent zBMI. There was also no significant effect of parent/guardian or adolescent ethnicity on BMI/zBMI. Thus, race and ethnicity were not included as covariates.

Parent/guardian BMI was negatively associated with total household income (r (242) = − 0.22, *p* < .01), parent/guardians’ combined education (r (243) = − 0.18, *p* < .01), and parent/guardians’ combined occupational prestige (r (236) = − 0.19, *p* < .01). Parent/guardian BMI was also positively associated with having any level of adult food insecurity (r (233) = 0.19, *p* < .01). When all three SES variables and the dummy coded food insecurity variables were entered into a multiple regression model (Table 3) along with parent/guardian age and sex, only very low food security remained significant in the model (β = 6.40, *p* < .05).

Adolescent zBMI was positively associated with having any level of (parent/guardian-reported) household food insecurity (r (233) = 0.16, *p* < .05). The subsequent multiple regression model, which controlled for adolescent age and sex, was not significant (*p* > .05).

### SES and reinforcement pathology

Adolescent RRV of HED food was negatively associated with household income (r (225) = − 0.15, *p* < .05). This relationship remained significant when controlling for participant hunger, food liking, dietary restraint, and reinforcing value of the alternative activity. The overall model was significant (Table 3). There were no significant associations between the reinforcing value of LED food and any measurement of economic position.

**Table 3:** Multiple Regression Analyses

Variables	B	SE	β	t	p	F	p	R^2^
Parent BMI						3.45	0.001	0.11
Parent Age	0.12	0.07	0.11	1.64	0.10			
Parent Sex	0.26	1.11	0.02	0.23	0.82			
Parents’ Education	−1.03	0.65	−0.13	−1.59	0.11			
Parents’ Occupation	−0.19	0.37	−0.04	−0.53	0.60			
Household Income	−1E-06	0.00	−0.11	−1.41	0.16			
Marginal Food Security	2.98	1.52	0.13	1.96	0.05			
Low Food Security	1.21	2.38	0.03	0.51	0.61			
Very Low Food Security	6.40	2.67	0.16	2.40	0.02			
Adolescent RRV of HED Food						9.91	0.000	0.19
Current Hunger	0.01	0.00	0.16	2.44	0.02			
Food Liking	0.03	0.01	0.30	4.73	0.00			
Dietary Restraint	−0.04	0.04	−0.07	−1.09	0.28			
RRV of Alternative	0.18	0.05	0.22	3.46	0.00			
Household Income	−4E-06	0.00	−0.15	−2.35	0.02			
Adolescent DD						3.35	0.002	0.10
Adolescent Age	1.12	0.36	0.18	3.10	0.00			
Adolescent Sex	−1.07	0.66	−0.11	−1.62	0.11			
Parents’ Education	−1.45	0.60	−0.23	−2.43	0.02			
Parents’ Occupation	0.08	0.29	0.02	0.26	0.79			
Marginal Food Security	0.38	1.19	0.02	0.32	0.75			
Low Food Security	1.11	0.81	0.05	1.38	0.17			
Very Low Food Security	1.67	2.26	0.04	0.74	0.46			
Adolescent RP						6.39	0.000	0.13
Adolescent Age	0.48	0.52	0.06	0.92	0.36			
Current Hunger	0.04	0.02	0.15	2.22	0.03			
Food Liking	0.07	0.02	0.21	3.13	0.00			
RRV of Alternative	0.50	0.21	0.16	2.41	0.02			
Socioeconomic Status	−4E-06	0.00	−0.21	−3.23	0.00			

*Note.* Multiple regression analyses are organized by dependent variable; all variables were entered into the models simultaneously

DD was associated with parent/guardians’ combined education (r (231) = − 0.24, *p* < .001), parent/guardians’ combined occupational prestige (r (225) = − 0.14, *p* < .05), and having any level of household food insecurity (r (221) = 0.14, *p* < .05). When all three of these variables along with adolescent age and sex were entered into the same model (Table 3) only parent/guardians’ combined education remained significant (β = − 1.45, *p* < .05). This model had a significant violation of the assumption of heteroskedastity and required adjustment of the standard errors.

In order to create a unified model, a composite SES variable was created by multiplying household income by parent/guardians’ education. RP was significantly associated with the composite SES variable (r (218) = 0.21, *p* < .01). This relationship remained significant after controlling for adolescent age, hunger, food liking, and RRV of the alternative activity (β = − 3.80^− 6^, *p* < .01) (Table 3).

## Discussion

This study extends the current literature on the relationship between SES and eating behavior. As has been shown in previous literature, our results demonstrate that SES is a confluence of related factors, all of which are related to food insecurity [9]. Our results further showed that parent/guardian BMI was related to parent/guardian food insecurity. Adolescent zBMI was associated with (parent/guardian-reported) household food insecurity. Despite this, RRV and DD, which interact to create RP, were unrelated to food insecurity. Instead, the RRV of HED foods was related to total household income and DD was related to parent/guardians’ education. These findings suggest that the relationship between SES and obesity risk is complex in terms of behavioral mechanisms.

Household income and parent/guardians’ education were negatively associated with the RRV of HED food and DD, respectively. Shah and colleagues [31] have posited that scarcity (i.e. having less than one believes they need) of any particular resource will result in an attentional bias toward that scarce resource. Because food and financial resources are intertwined for humans [2], any decrease in abundance of one may be associated with increased motivation for the other. The graded relationship observed here suggests that the fewer household financial resources, the higher the reinforcing value for HED foods among the adolescents. However, this was not the case for LED foods, the RRV of which was unrelated to any of our measures of SES. This is somewhat unexpected because LED foods tend to be the scarcest type of food in impoverished homes [22]. However, previous work has shown that scarce financial resources tend to motivate an intake of more energy, rather than a scarce type of food [2]. Previous work has also shown a relationship between SES and DD in adults [25]. Further, evidence suggests that the uncertain environment associated with lower SES may disincentivize delaying gratification and thereby discourage children from developing this ability [27].

Our results deviate from one another when it comes to adolescent behavior and zBMI. We found support for our second hypothesis, that food insecurity is related to parent/guardian BMI. This was also the case for adolescent zBMI, though this was not significant after controlling for covariates. Past work has shown that adults in the home tend to shield their children from food insecurity, which may explain the discrepancy in this variable between the parent/guardians and the adolescents [23]. Although food insecurity and income are related to one another, they are distinct constructs; and while income has a wide range of values, food insecurity is more restricted and represents a relatively severe, and uncommon, form of scarcity in the United States [9]. Although we cannot assume causation from these cross-sectional data, we suspect that small changes in income and education may affect motivation and inhibition in an adolescent, which may accumulate over time to increase the risk for obesity [3]. By contrast, food insecurity, which has a large effect on the food in a household (i.e. favoring more energy density) will have a stronger, and more immediate, effect on body size [22]. When occurring together, we suspect that a shift in energy density in the household food supply related to food insecurity and changes in motivation and inhibitory control related to income and education may synergize to increase the energy intake of an adolescent in an impoverished home. However, more research, particularly longitudinal assessment, is needed to fully explore this hypothesis.

The relation between SES and adolescent behavior but not zBMI may indicate an opportunity for intervention before obesity develops. Our results suggest that different aspects of SES may affect these mechanisms in distinct ways, which can further help to inform interventions in this area. For example, increasing access to healthy (i.e. LED) foods in impoverished areas may improve healthy eating and diet quality. While increasing access to healthy food is crucial for health equity, our results suggest that increasing access to education in the United States broadly, as well as increasing food purchasing power might create a greater change in eating behavior for the adolescents in a low SES home.

This study has many strengths. Previous work has shown a relationship between SES and both RRV and DD in adults, but no studies have examined these relationships in adolescents. This study included a large sample of adolescents with objective measurements of zBMI, RRV, and DD. Past research has often focused on RRV and DD separately, but this study examines both, which allows us to draw conclusions about both motivational and inhibitory mechanisms, and their relationship with SES, simultaneously. Despite the strengths of this study, all of our analyses must be considered in the context of the sample that was collected for this study and the limitations that this imposes on our ability to draw conclusions. The parent surveys in this study were largely answered by the adolescents’ biological mothers (77%), but some biological fathers, grandparents, and other guardians were also included in the sample. These caregivers may have interpreted and answered questions in different ways than the biological mothers who made up the majority of the sample, which could have limited our ability to see important relationships in these data. Although we found no association between parent/guardian sex and the variables of interest, the lack of consistency in parent/guardian participation could have affected the results of this study in unknown ways. Though this sample had a range of income and education levels, it skewed toward the higher end of these spectrums and had limited racial/ethnic diversity. The results shown here may not generalize to a more diverse population or one with more people living near the poverty line. Additionally, because the overall goal for the broader longitudinal study was to examine obesity risk factors, this was a sample without obesity. However, the first study appointment, which was used for screening purposes, included a small number of adolescents with obesity (*N* = 4). Thus, the analysis of food insecurity, DD and BMI/zBMI included more families, as well as adolescents with obesity, compared to those with RRV data. The largely non-obese sample as well as the smaller N for the RRV data may have limited our ability to examine any potential relationship between food insecurity and RRV. We suggest that our findings should be taken as evidence for the need for further investigation into these topics using samples that better represent the economic divide in the United States as well as more racial/ethnic diversity.

## Conclusions

When taken together, our results suggest that household income, parent/guardian education, and food insecurity are interrelated, but separate, constructs that may exert differential effects on behavior and obesity risk. Among adolescents, our results suggest that low income and low parent/guardian education, when occurring together, will increase RP. Separately, in the most severe cases, families with food insecurity, both parent/guardians and adolescents showed higher BMIs. This analysis did not find evidence for a pathway from low SES to zBMI in the adolescents, but we did observe this pathway in the parent/guardians. Further, the associations here are small, but small changes in behavior over a long period of time have a large effect on obesity risk, and we suspect that these small shifts in RP will have an effect both on the adolescents’ emerging eating behaviors as well as their risk for adult obesity. Future research should continue this investigation by examining the effects of low SES on RP over time as well as examining them in an experimental context.

## Supplementary Information

**Additional file 1.**

## Data Availability

The datasets generated and analyzed during the current study are not publicly available at the time of publication because this is an ongoing longitudinal study and participants are still enrolled in the study. However, deidentified data are available from the corresponding author upon reasonable request.

## Abbreviations

ANOVA: Analysis of Variance
BMI: Body Mass Index
DD: Delay Discounting
DEBQ: Dutch Eating Behavior Questionnaire
HED: High Energy Density
LED: Low Energy Density
RP: Reinforcement Pathology
RRV: Relative Reinforcing Value
SES: Socioeconomic Status
SNAP: Supplemental Nutrition Access Program
zBMI: z-scored Body Mass Index
