# Supplementary material for: Having less and wanting more: an investigation of socioeconomic status and reinforcement pathology
Source: BMC Public Health. 2021 Feb 25;21:402. doi: 10.1186/s12889-021-10430-7 (PMC7905857; doi:10.1186/s12889-021-10430-7)

***Hispanic or Latino: “****A person of Mexican, Puerto Rican, Cuban, South or Central American, or other Spanish culture of origin, regardless of race. The term “Spanish origin” can be used in addition to “Hispanic or Latino.”*

1. Do you consider your child to be Hispanic or Latino? (See definition above)

🞏 **YES** 🞏 **NO**

1. What race does your child consider themselves to be? (Check All that Apply)

- American Indian or Alaskan Native 🞏 Native Hawaiian or Other Pacific Islander
- Asian 🞏 White or Caucasian
- Black or African American 🞏 Other - please specify: _______________________________

1. The child spends time in:
   1. One household
   2. Two households (with the majority of time spent **primarily** in one household)
   3. Two households (equal time spent in both households)
2. Which of the following currently live in the **primary** household of the child? (Check all that apply)

🡪If the child spends equal time in two households, please consider both homes **“primary.”**

- Biological Father
- Stepfather
- Biological Mother
- Stepmother
- Legal Guardian
- OTHER: **please specify**_______________________

1. What is **YOUR** relationship to the child in this study **(Parent/Guardian #1)?**
   1. Do you currently live in the **primary** household of the child?

🡪If the child spends equal time in two households, please consider both homes **“primary.”**

- - - YES 🞏 NO
  1. Which of the following is **YOUR** relationship to the child?
- Biological Father
- Stepfather
- Biological Mother
- Stepmother
- Legal Guardian
- OTHER: **please specify** _______________________

1. Total household income for **YOUR** home is derived from (check all that apply)
   - One income 🞏 Public assistance
   - Two incomes 🞏 Child support/alimony
   - Unemployment 🞏 OTHER: **please specify** ________________________________­­­­­_____
2. **YOUR** Total household income last calendar year (including child support, alimony, etc):
   1. Under $9,999 6. $ 90,000 – 109,999
   2. $10,000 – 29,999 7. $110,000 – 139,999
   3. $30,000 – 49,999 8. $140,000 – 179,999
   4. $50,000 – 69,999 9. $180,000 – 199,999
   5. $70,000 – 89,999 10. Over $200,000
3. What is **YOUR** marital status?
   - 1. Single
     2. Married
     3. Divorced
     4. Living with significant other **and** sharing financial resources
4. **YOUR** highest level of education completed:
   - 1. Less than seventh grade
     2. Junior high (9^th^ grade)
     3. Some high school (10^th^ or 11^th^ grade)
     4. Completed high school
     5. Some college and/or completed vocational training
     6. Completed college or university (2 or 4-year degree)
     7. Completed graduate degree (beyond bachelor degree)
5. **YOUR** Current employment status:
   - 1. Working full time 4. Unemployed
     2. Working part time 5. Homemaker
     3. Laid off: ________________ 6. Retired ________________________________________

(DATE) (PREVIOUS INDUSTRY AND POSITION)

1. **YOUR** Occupation (Specific Name of Industry and Position): _____________________________________
2. Do you consider **YOURSELF** to be Hispanic or Latino? (*see definition on page 1*)

🞏 YES 🞏NO

1. What race do you consider **YOURSELF** to be? (Check All that Apply)
   - American Indian or Alaskan Native 🞏Native Hawaiian or Other Pacific Islander
   - Asian 🞏 White or Caucasian
   - Black or African American 🞏 Other - please specify:_________________________
2. **YOUR** *current* stature most closely resembles which of the following sketches? (please circle one)


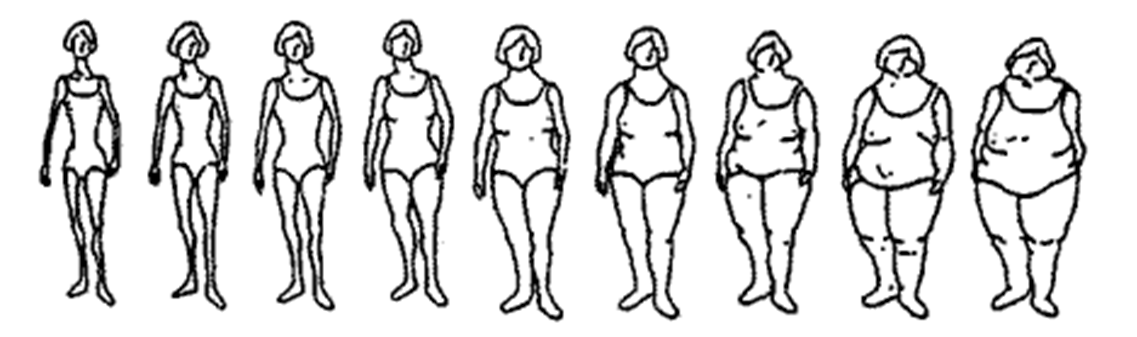


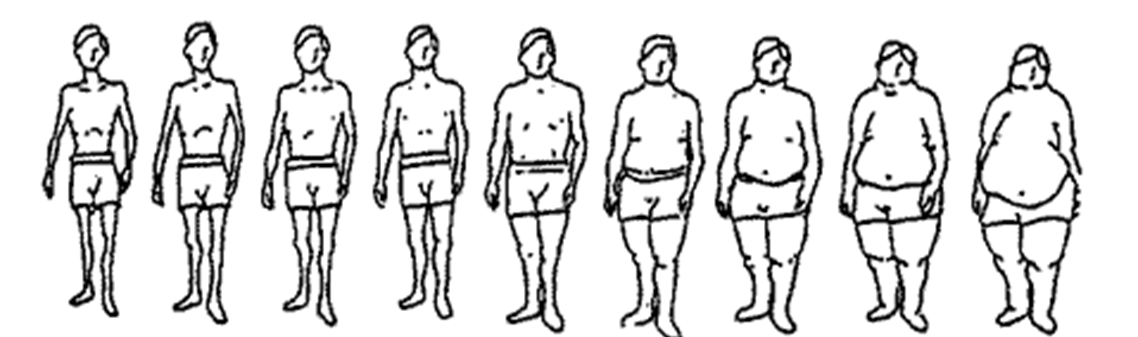


**IF YOU ARE THE ONLY ADULT CAREGIVER** FOR THIS CHILD, PLEASE SKIP ALL OF THE FOLLOWING QUESTIONS.

1. Who would be considered the child’s **Parent/Guardian #2?**

- Biological Father
- Stepfather
- Biological Mother
- Stepmother
- I am the only caregiver for this child
- Another Legal Guardian
- OTHER: **please specify**_______________________

1. Highest level of education completed by child’s **Parent/Guardian #2**:
   - 1. Less than seventh grade
     2. Junior high (9^th^ grade)
     3. Some high school (10^th^ or 11^th^ grade)
     4. Completed high school
     5. Some college and/or completed vocational training
     6. Completed college or university (2 or 4-year degree)
     7. Completed graduate degree (beyond bachelor degree)
2. Current employment status of child’s **Parent/Guardian #2**:
3. Working full time 4. Unemployed
4. Working part time 5. Homemaker
5. Laid off: ________________ 6. Retired ________________________________________________

(DATE) (PREVIOUS INDUSTRY AND POSITION)

1. Occupation of child’s **Parent/Guardian #2**: (Specific Name of Industry and Position): __________________________
2. Do you consider child’s **Parent/Guardian #2** to be Hispanic or Latino? (*see definition on page 1*)

🞏 YES 🞏NO

1. What race do you consider child’s **Parent/Guardian #2** to be? (Check All that Apply)
   - American Indian or Alaskan Native 🞏Native Hawaiian or Other Pacific Islander
   - Asian 🞏 White or Caucasian
   - Black or African American 🞏 Other - please specify:_________________________
2. Which sketch most closely resembles the *current* stature of child’s **Parent/Guardian #2**? (please circle one)


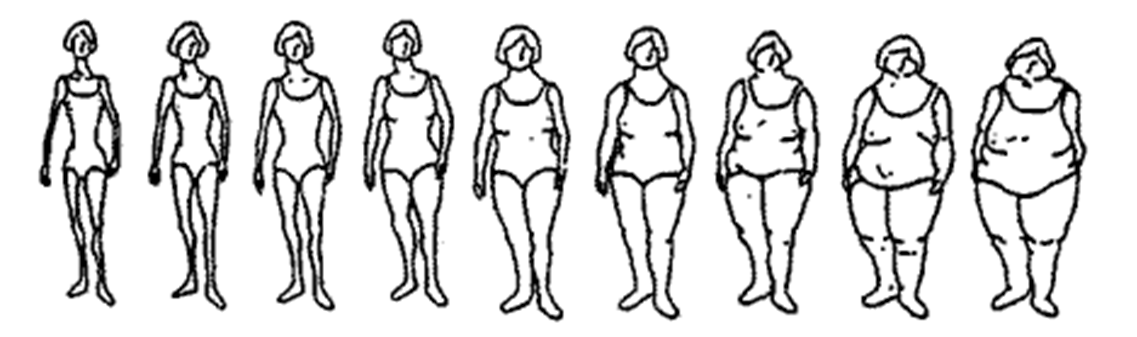


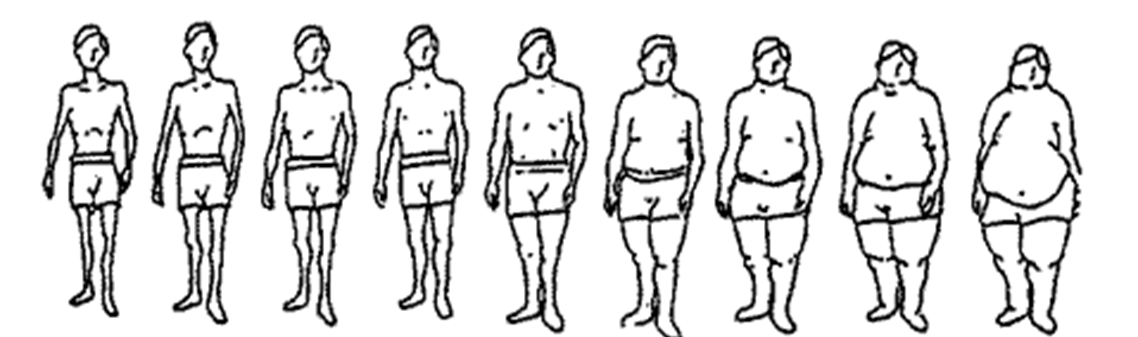


COMPLETE **#22-25** IF YOUR CHILD SPENDS TIME IN MORE THAN ONE HOUSEHOLD.

1. What other adult caregiver does your child live with part of the time**?** (**Adult #3)**

- Biological Father
- Stepfather
- Biological Mother
- Stepmother
- I am the only caregiver for this child
- Another Legal Guardian
- OTHER: **please specify**_______________________

1. Which sketch most closely resembles the *current* stature of **Adult #3**? (please circle one)


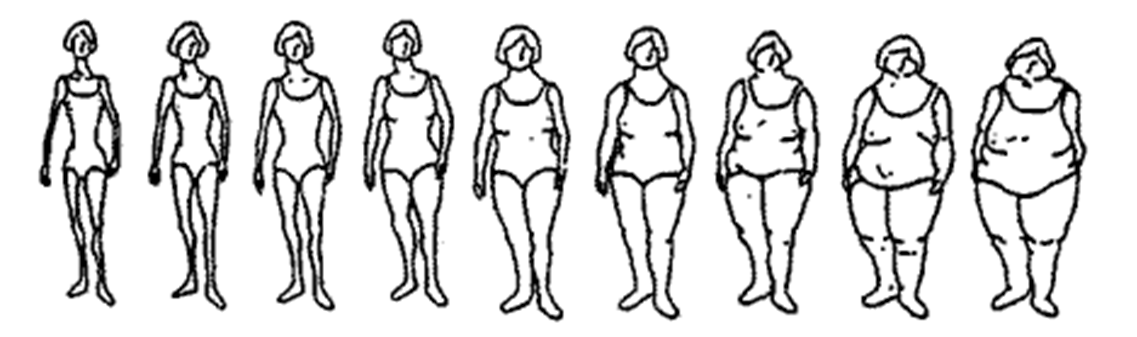


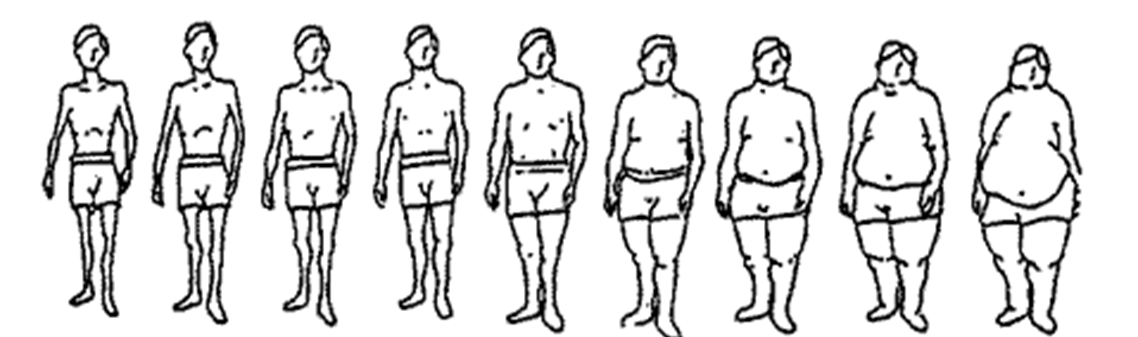


1. What other adult caregiver does your child live with part of the time**?** (**Adult #4)**

- Biological Father
- Stepfather
- Biological Mother
- Stepmother
- I am the only caregiver for this child
- Another Legal Guardian
- OTHER: **please specify**_______________________

1.
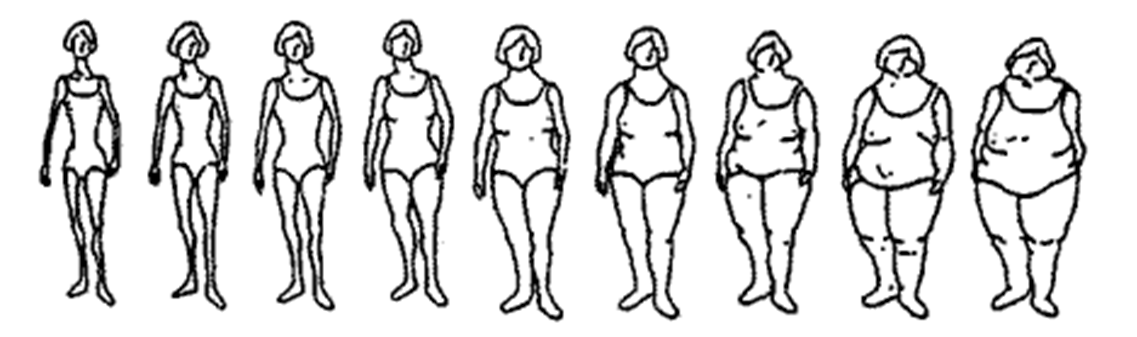
Which sketch most closely resembles the *current* stature of **Adult #4**? (please circle one)


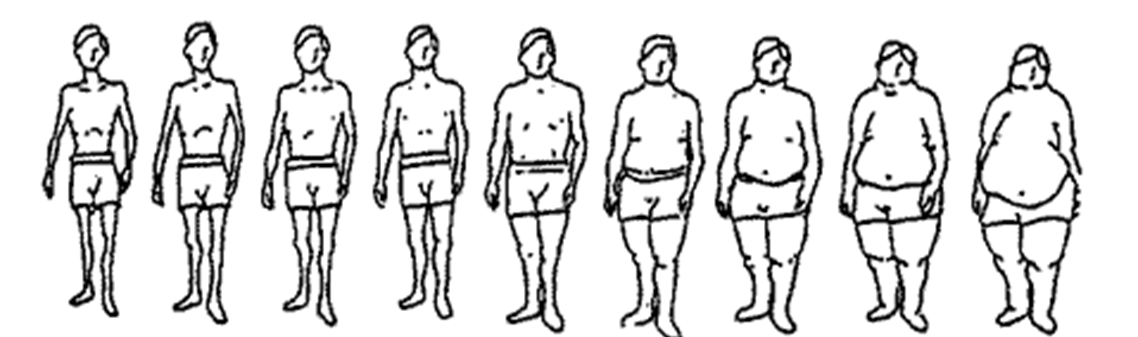

Supplement: Supplementary file 1 — Additional file 1. [file 12889_2021_10430_MOESM1_ESM.docx]
